# A core outcome set for cranioplasty following stroke or traumatic brain injury - The COAST study

**DOI:** 10.1016/j.bas.2025.104288

**Published:** 2025-06-01

**Authors:** H. Mee, T.K. Korhonen, A.M. Castaño-Leon, A. Adeleye, J. Allanson, F. Anwar, I.D. Bhagavatula, K. Bond, C. Clement, A.M. Rubiano, K. Grieve, G. Hawryluk, A. Helmy, S. Honeybul, C. Iaccarino, A. Lagares, H. Marcus, N. Marklund, S. Muehlschlegel, N. Owen, M. Paul, V. Pomeroy, D. Shukla, F. Servadei, E. Viaroli, E. Warburton, A. Wells, I. Timofeev, C. Turner, G. Whiting, P. Hutchinson, A. Kolias

**Affiliations:** a Division of Neurosurgery, Department of Clinical Neurosciences, University of Cambridge, Cambridge, United Kingdom; b Division of Rehabilitation Medicine, Department of Clinical Neurosciences, Cambridge University Hospital NHS Foundation Trust, Cambridge, United Kingdom; c NIHR Global Health Research Group on ABSI, University of Cambridge, Cambridge, United Kingdom; d Neurocenter, Neurosurgery, Oulu University Hospital & University of Oulu, Oulu, Finland; e Department of Neurosurgery, Instituto de Investigación Sanitaria Hospital 12 de Octubre, Hospital Universitario 12 de Octubre, Madrid, Spain; f Division of Neurological Surgery, Department of Surgery, College of Medicine, University of Ibadan, And University College Hospital, Ibadan, Nigeria; g Division of Anaesthesia, University of Cambridge, Cambridge, United Kingdom; h Department of Neurosurgery, National Institute of Mental Health and Neurosciences, Bangalore, India; i Bristol Trials Centre, Bristol Medical School, Bristol, United Kingdom; j Neurosciences and Neurosurgery, INUB/Meditech Research Group, Cali, Colombia; k Division of Neurosurgery, Department of Clinical Neurosciences, Cambridge University Hospital NHS Foundation Trust, Cambridge, United Kingdom; l Section of Neurosurgery, University of Manitoba, MB, Canada; m Department of Neurosurgery, Royal Perth Hospital, Perth, Australia; n School of Neurosurgery, Department of Biomedical, Metabolic and Neural Sciences, University of Modena and Reggio Emilia, Italy; o Neurosurgery Unit, University-Hospital of Modena, Modena, Italy; p Neurosurgery Unit, AUSL RE IRCCS, Reggio Emilia, Italy; q Department of Neurosurgery, National Hospital for Neurology and Neurosurgery, Queen Square, London, United Kingdom; r Department of Brain Repair and Rehabilitation, UCL Queen Square Institute of Neurology, Queen Square, London, United Kingdom; s Department of Clinical Sciences Lund, Neurosurgery, Lund University, And Skane University Hospital, Lund, Sweden; t University of Massachusetts Medical School, MA, United States; u Adelaide University, Adelaide, Australia; v School of Health Sciences, University of East Anglia, United Kingdom and National Institute Health Research HelathTech Research Centre in Brain Injury, Norwich, United Kingdom; w Humanitas Clinical and Research Center, Istituto di Ricovero e Cura a Carattere Scientifico (IRCCS), Milan, Rozzano, Italy; x Department of Biomedical Sciences, Humanitas University, Pieve Emanuele, Milan, Italy; y Division of Stroke Medicine, Department of Clinical Neurosciences, Cambridge University Hospital NHS Foundation Trust, Cambridge, United Kingdom; z Department of Neurosurgery, Royal Adelaide Hospital, Adelaide, Australia; aa ValleSalud Clinical Network, Cali, Colombia

**Keywords:** Cranioplasty, Outcomes, Consensus, Core outcome set, Rehabilitation

## Abstract

**Introduction:**

There is substantial heterogeneity in the reporting of outcomes in the global cranioplasty literature. This study aimed to establish a core outcome set (COS) for cranioplasty after decompressive craniectomy for stroke or traumatic brain injury.

**Methodology:**

The scope was defined according to the criteria recommended by the Core Outcome Measures in Effectiveness Trials (COMET) Initiative. Phase 1 focused on outcome gathering through a systematic review and a qualitative study. Phase 2 focused on consolidation and consensus of outcomes through a two-round Delphi survey and consensus meeting. Participants from the four stakeholder groups (1. patients and/or relatives; 2. Surgeons, 3. physicians (non-surgeons), 4. Nurses, allied health professionals, and researchers) individually scored all outcomes on a 9-point Likert scale. Variables that did not reach the predefined consensus threshold for COS inclusion or exclusion were voted upon at the final consensus meeting.

**Results:**

In total, 208 verbatim outcomes were consolidated into 56 domains. A total of 153 participants completed round 1, with 45 additional outcomes suggested for inclusion. Following rationalisation, four were included in round 2. A total of 109/153 participants (71 %) from 16 countries completed Round 2 and re-scored all 60 outcomes (56 original + 4 additional). Nine outcomes were voted in, and 12 were excluded from the Delphi. The remaining 39 were discussed at a consensus meeting with 11 voted in. The final COS included 20 outcomes (12 + 8) across four domains: life impact, pathophysiological manifestations, resource use/economic impact, and mortality.

**Conclusion:**

COAST COS covers key cranioplasty outcomes, as assessed by international stakeholders, including surgical, medical, rehabilitation, and nursing professionals, as well as patients and their relatives. Future implementation will aid in the standardisation of outcomes and facilitate the development of cranioplasty-specific outcome measures, aiding between-study comparisons and improving the relevance of trial findings to healthcare professionals and patients.

## Highlights

• The COAST study established a core outcome set (COS) of 20 outcomes for cranioplasty.
• The final COS covers four key domains: life impact, pathophysiological manifestations, resource use/economic impact, and mortality.
• Important life impact outcomes include overall function, functional independence, cognition, and cosmesis.
• Complications were a major focus in pathophysiological manifestation, highlighting the significant burden of complications associated with cranioplasty.
• COAST COS aims to standardise outcome reporting in cranioplasty research and clinical practice.

## 1 Introduction

### 1.1 Background and objectives

Cranial reconstruction of skull bone defects, also known as cranioplasty, is performed to restore protective, aesthetic, and functional properties of the cranium. Between 2014 and 2019, the number of cranial decompressive procedures in the UK increased by approximately 12 %, following recent high-level evidence on the effectiveness of decompressive craniectomy following traumatic brain injury (TBI) and stroke (Hutchinson et al., 2016; Vahedi et al., 2007). This has likely translated into an increased number of cranioplasty procedures and has accelerated research interest over the past decade (Mee et al., 2023a).

Cranioplasty is often associated with salutary physiological and functional changes, improved cerebral metabolism (Halani et al., 2017; Lilja-Cyron et al., 2019; Winkler et al., 2000) postoperatively, as well as neurological outcomes measured through various rehabilitation indices (Mele et al., 2022) and neuropsychological testing (Cola et al., 2018; Malcolm et al., 2018); postoperative complications are common. The complication rates range from 4 % to 45 %, depending on the definition used, and include surgical site infections, autologous bone flap resorption, and hydrocephalus (Malcolm et al., 2016). They often require repeat surgery and prolonged hospital admission, all of which affect a patient's recovery trajectory and quality of life.

Despite the wide range of published literature on cranioplasty, the lack of high-quality evidence has hindered the development of evidence-based guidelines. The existing literature, which mainly consists of single-centre, retrospective studies (Mee et al., 2023a; Malcolm et al., 2018; Cerveau et al., 2023), shows significant variation in practice patterns, a wide range of complication rates, and considerable variation in clinical definitions, data collection, and outcome reporting, ultimately resulting in significant outcome differences. Such heterogeneity impedes meta-analyses and complicates the interpretation of the evidence base, posing substantial barriers to establishing an evidence-based approach to cranioplasty care and research, such as determining the optimal time to replace the bone flap and the optimal material in relation to neurological recovery and complications. Optimising best practices, standardised data, and outcome collection are prerequisites for addressing some of these concerns.

We aimed to produce a COS for cranioplasty outcome collection and reporting following stroke or TBI in a two-phase process defined according to the Core Outcome Measures in Effectiveness Trials (COMET) Initiative criteria (Williamson et al., 2017). In Phase 1, we gathered commonly collected outcomes through a systematic literature review (Mee et al., 2023a), and qualitative study which was followed by Phase 2: a stakeholder Delphi process and consensus meeting to establish a consensus on the outcome inclusion for a COS. Here, we report a final consensus-based COS to aid in the standardisation of cranioplasty outcome collection and reporting.

### 1.2 Scope

**Fig. 1:**
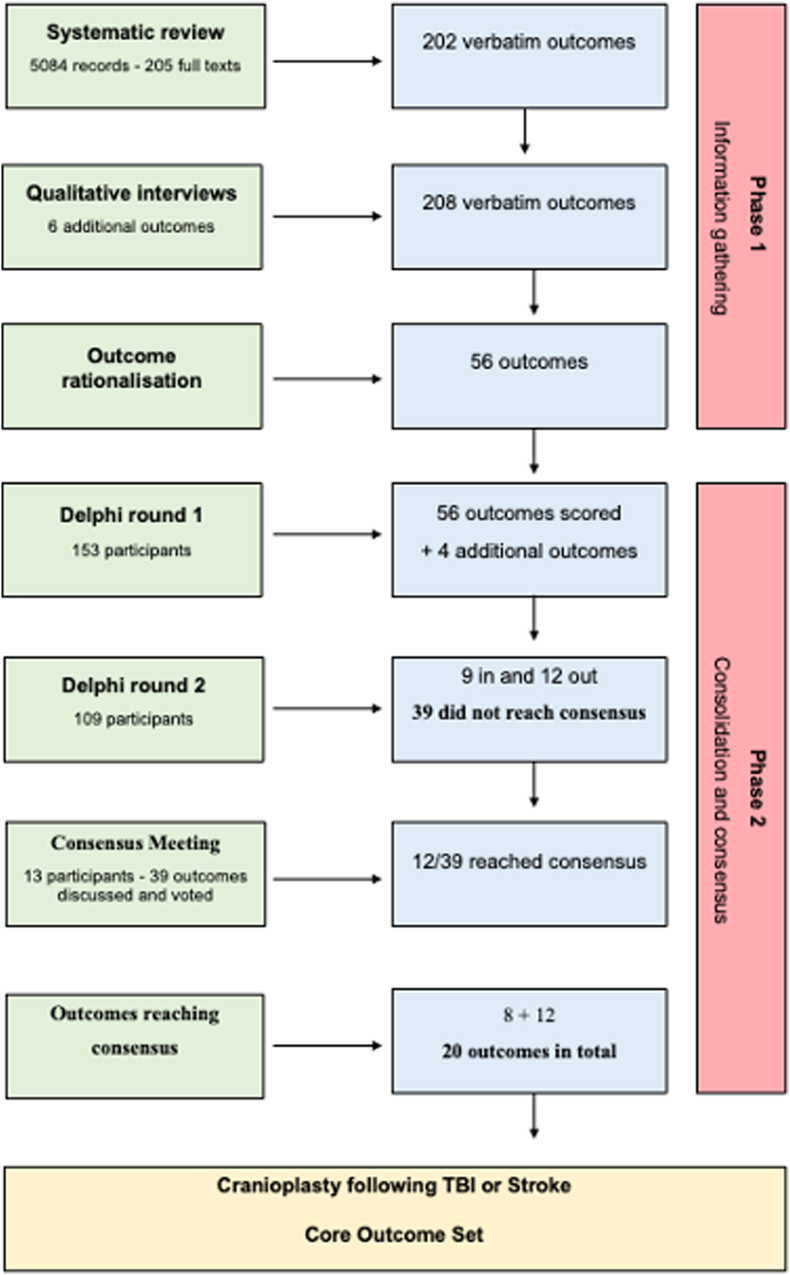
COAST study flow chart.

The scope of the study was defined according to the criteria recommended by the COMET Initiative (Williamson et al., 2017). The health condition included adult patients (aged 16 years or older) who had undergone decompressive craniectomy secondary to TBI or stroke and were either awaiting or had undergone cranioplasty, with the health intervention being cranioplasty. Guidelines for decompressive craniectomy (DC) in the context of stroke typically identify middle cerebral artery ischemia as the primary indication for the procedure. However, in clinical practice, the scope has expanded to encompass other pathologies classified under stroke, and this Core Outcome Set (COS) reflects this evolution in clinical practice. An adapted Outcome Measures in Rheumatology (OMERACT) filter 2.0 was used to aid classification into core areas: life impact, pathophysiological manifestations, resource and economic impact, and mortality/survival. This was done to ensure its relevance to both clinical practice and research. The study was conducted using the methodological process outlined in the COMET Handbook (Williamson et al., 2017) and conformed to the standards guiding COS development (COS-STAD) (Kirkham et al., 2017) and the COMET protocol standards (COS-STAP) (Kirkham et al., 2019) (Fig. 1).

## 2 Materials and methods

### 2.1 Protocol registration

The study protocol was published separately (Mee et al., 2023b) and registered on the Core Outcome Measures in Effectiveness Trials (COMET) database. The Core Outcome Set-STAndards for Reporting (COS-Star) guidelines were adhered to in the reporting of this COS (Kirkham et al., 2016).

### 2.2 Stakeholders and COS development team

Participants in the Delphi study were assigned to one of four stakeholder groups: 1. patients and/or relatives; 2. Surgeons, 3. physicians (non-surgeons), and 4. Nurses, allied health professionals, and researchers. These stakeholder groups represent key personnel in the acute and long-term management and rehabilitation of patients who have undergone or will undergo cranioplasty.

An international steering committee was formed to oversee the development of the COS, which included representation from stakeholder groups and a project management team that managed the practical aspects of COS development and reported to the steering committee with any methodological or study conduct concerns.

### 2.3 Patient and public involvement

Patient and family member involvement was a critical methodological consideration throughout the study. There was patient representation on the steering committee and one stakeholder group was specifically for patients and relatives. Their involvement was in both phases 1 and 2 of the study and in the final consensus meeting.

### 2.4 Study phases – information sources

#### 2.4.1 Phase 1: information gathering

A systematic review, with the aim of understanding the outcomes reported in the published literature, was conducted and published separately (Mee et al., 2023a). In total, 202 verbatim outcomes were extracted from 205 papers. In addition to this review, a qualitative study involving four stakeholder groups was conducted to ensure a comprehensive list of relevant outcomes, resulting in six additional outcomes, giving a total of 208 identified outcomes (verbatim outcomes). As there was considerable overlap between some outcomes, they were rationalised into a list of 56 by the working group and reviewed by a COMET team member to maintain their validity.

The Delphi questionnaire was then developed to include the 56 voting outcomes within the Delphi.

#### 2.4.2 Phase 2: consolidation and consensus

##### 2.4.2.1 Delphi Survey

**Fig. 2:**
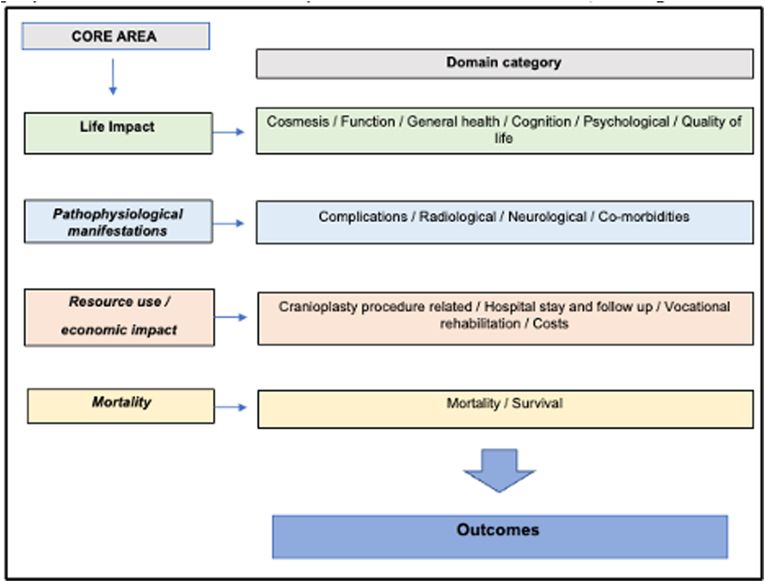
Adapted OMERACT filter 2.0 for cranioplasty COS.

The Delphi process was conducted iteratively using the Delphi method (Jones and Hunter, 1995). The 56 outcomes from Phase 1 were grouped into outcome domains and categorized into one or more of the OMERACT filter 2.0 core areas (life impact, pathophysiological manifestations, resource use/economic impact, and mortality) (Boers et al., 2014) (Fig. 2). These were then formulated into a questionnaire (Appendix 1) used to populate the Delphi Survey. The survey was conducted using the DelphiManager platform (blinded for reviewer). The survey was circulated widely via professional networks, patient forums, and social media to all those involved in cranioplasty care. All the survey participants categorized themselves into one of the four stakeholder groups.

##### 2.4.2.2 Outcome scoring and consensus process

The Delphi method consisted of two rounds of voting, followed by an online consensus meeting. In the first round, which was open for one month, all participants scored the importance of each individual outcome using a 9-point Likert scale: 1–3 – limited importance, 4–6 – important but not critical, 7–9 – critically important. Participants could also put additional outcomes forward for consideration in Round 2 scoring. Following Round 1, the results were analysed, comments were collated, and the proposed additional outcomes were rationalised and voted on for Round 2 scoring by the working group and steering committee.

In round 2, participants were asked to re-score the outcome using the same scoring criteria. Participants were able to view the aggregated scores and feedback from their stakeholder group from round 1, and could change their scores if they wished. Following Round 2, the predefined consensus definition (Table 1) was applied, and each outcome domain was scored accordingly. Outcomes that reached ‘consensus in’ were directly included in the consensus-based COS, those reaching ‘consensus out’ were excluded from the COS, and those in the ‘no consensus’ category were taken forward for discussion and voting for COS inclusion in the final consensus meeting.

**Table 1:** Consensus definitions for Delphi survey.

**Consensus in**	70 % or more participants scoring the outcome "7 to 9″ AND fewer than 15 % scoring "1 to 3″
**Consensus out**	50 % or less, scoring the outcome as 7 to 9
**No consensus**	Any outcome not reaching the criteria for consensus 'in' or ‘out'

### 2.5 Consensus meeting

Participants from each stakeholder group were invited to a consensus e-meeting held on the January 11, 2023 on the Zoom platform (Zoom Video Communications, Inc., San José, CA, USA) to discuss the survey results and vote on the outcomes that did not reach a consensus on the second round of the Delphi survey. The consensus meeting included members from each of the four stakeholder groups and was chaired by a neurosurgical consultant independent of the study team. Each outcome that did not reach consensus in the second Delphi survey round was presented in the meeting and discussed before voting. The voting options for each outcome were 'consensus in, consensus out, and abstain’, with a 70 % consensus required for inclusion in the COS.

### 2.6 Ethics and consent

This study was approved by the UK HRA Ethics Committee (blinded to the reviewer). An international steering committee oversaw the development of the COS. All study participants provided informed consent, and their data were processed by the study team at the beginning of the Delphi process (round 1). All data was processed in accordance with the UK data protection regulations.

## 3 Results

### 3.1 Phase 2: consolidation and consensus

#### 3.1.1 Delphi results

One hundred and ninety-three participants were registered on the online Delphi platform. Of these, 153 (79 %) completed Round 1 (Table 2). All 153 participants from round 1 were invited to participate in round 2, of whom 109 (71 %) participants from 16 countries completed the second round (Table 3).

**Table 2:** Delphi Survey respondent stakeholder distribution.

Delphi participants	Round 1	Round 2
**Patient/Relative**	16 (10 %)	11 (10 %)
**Surgeon**	70 (46 %)	50 (46 %)
**Physician (non-surgeon)**	37 (24 %)	29 (27 %)
**Nurse, AHP, Researcher**	30 (20 %)	19 (17 %)
**Total**	**153**	**109**

**Table 3:** Country of origin of participants completing Delphi survey round 2.

**Australia**	3	*Ireland*	1
**Brazil**	1	*Italy*	4
**Canada**	1	*Other*	4
**Cyprus**	1	*Romania*	1
**Finland**	1	*Spain*	11
**France**	1	*Sweden*	2
**Germany**	3	*United Kingdom*	52
**India**	14	*United States*	17

##### 3.1.1.1 Round 1

All the 56 outcomes were scored. The participants suggested 45 additional outcomes for Round 2 scoring. Of these, 10/45 (22 %) were not considered outcomes, and 22/45 (49 %) were either direct or indirect replications of an already proposed outcome. Therefore, 13/45 (29 %) additional outcomes were put forward to the steering committee to vote for inclusion in Round 2, of which 4/13 (30 %) were included (Appendix 2).

##### 3.1.1.2 Round 2

The 109 participants who completed Round 2 re-scored all 60 outcomes (56 original and 4 additional). According to the predefined consensus definitions (Table 1), 9/60 (16 %) outcomes met the COS inclusion criteria and 12 (20 %) were excluded (Appendix 2). The remaining 39 (72 %) outcomes did not reach a consensus and were included in the consensus meeting agenda for further discussion and voting for COS inclusion.

A complete summary of all the scores from both rounds of the Delphi survey is provided in Appendix 5.

### 3.2 Consensus meeting

Thirteen participants attended a meeting with representation from each stakeholder group. Of the 39 variables voted on, 12 (31 %) fulfilled the inclusion criteria and were accepted in the COS (Table 4). The voting response for each outcome varied from 92 to 100 %.

**Table 4:** Consensus meeting voting results.

Proposed Outcome	Consensus meeting voting
Overall cosmetic outcome following cranioplasty	In: 10 (77 %) Out: 3 (23 %) Abstain: 0 (0 %)
Patient satisfaction of cosmetic outcome	In: 11 (85 %) Out: 1 (8 %) Abstain: 1 (8 %)
Motor function following cranioplasty	In: 8 (62 %) Out: 4 (31 %) Abstain: 1 (8 %)
Effect of pain on function	In: 1 (8 %) Out: 12 (92 %) Abstain: 0 (0 %)
Changes in overall physical health following cranioplasty	In: 3 (23 %) Out: 9 (69 %) Abstain: 1 (8 %)
Potential changes in bladder control following cranioplasty	In: 1 (8 %) Out: 9 (69 %) Abstain: 3 (23 %)
Potential change in bowel control following cranioplasty	In: 1 (8 %) Out: 9 (69 %) Abstain: 3 (23 %)
Impact of cranioplasty on overall patient well-being	In: 3 (38 %) Out: 8 (62 %) Abstain: 2 (15 %)
The effect of cranioplasty on overall cognition	In: 11 (85 %) Out: 1 (8 %) Abstain: 1 (8 %)
The effect of cranioplasty on executive functioning	In: 5 (38 %) Out: 7 (54 %) Abstain: 1 (8 %)
The effect of cranioplasty on memory	In: 4 (31 %) Out: 9 (69 %) Abstain: 0 (0 %)
The effect of cranioplasty on attention	In: 6 (46 %) Out: 7 (54 %) Abstain: 0 (0 %)
The effect of cranioplasty on orientation	In: 7 (54 %) Out: 6 (46 %) Abstain: 0 (0 %)
The effect that a cranioplasty may have on the overall mental health/well-being of a patient	In: 10 (77 %) Out: 3 (23 %) Abstain: 0 (0 %)
The effect of cranioplasty on depression and anxiety	In: 3 (23 %) Out: 10 (77 %) Abstain: 0 (0 %)
The impact cranioplasty has on a patient's social outcome	In: 6 (46 %) Out: 7 (54 %) Abstain: 0 (0 %)
Wound/soft tissue related issue	In: 10 (83 %) Out: 2 (17 %) Abstain: 0 (0 %)
Intra-cranial haematoma	In: 12 (100 %) Out: 0 (0 %) Abstain: 0 (0 %)
Extra-cranial haematoma collections	In: 10 (77 %) Out: 3 (23 %) Abstain: 0 (0 %)
Hydrocephalus	In: 11 (92 %) Out: 1 (8 %) Abstain: 0 (0 %)
Graft specific complications	In: 12 (100 %) Out: 3 (23 %) Abstain: 0 (0 %)
Medical – systemic complications	In: 5 (42 %) Out: 7 (58 %) Abstain: 0 (0 %)
Cerebrovascular events	In: 8 (67 %) Out: 4 (33 %) Abstain: 0 (0 %)
Pain - scalp	In: 1 (8 %) Out: 11 (92 %) Abstain: 0 (0 %)
Headache	In: 4 (33 %) Out: 8 (67 %) Abstain: 0 (0 %)
Bone resorption	In: 11 (85 %) Out: 2 (15 %) Abstain: 0 (0 %)
Bone necrosis	In: 6 (46 %) Out: 4 (31 %) Abstain: 3 (23 %)
Intra-cranial haematoma	In: 6 (46 %) Out: 7 (54 %) Abstain: 0 (0 %)
Changes in level of consciousness	In: 12 (100 %) Out: 0 (0 %) Abstain: 0 (0 %)
Muscle function	In: 3 (25 %) Out: 9 (75 %) Abstain: 0 (0 %)
Patient's ability to swallow and communicate	In: 3 (25 %) Out: 8 (67 %) Abstain: 1 (8 %)
Sleep	In: 3 (25 %) Out: 9 (75 %) Abstain: 0 (0 %)
Physical symptoms of neurological nature	In: 6 (50 %) Out: 6 (50 %) Abstain: 0 (0 %)
Patients' co-morbidities	In: 6 (46 %) Out: 6 (46 %) Abstain: 1 (8 %)
Any repeat interventions	In: 9 (75 %) Out: 2 (17 %) Abstain: 1 (8 %)
Implant failure	In: 8 (67 %) Out: 3 (25 %) Abstain: 1 (8 %)
Timing of procedure	In: 10 (83 %) Out: 2 (17 %) Abstain: 0 (0 %)
Length of hospitalisation	In: 6 (50 %) Out: 6 (50 %) Abstain: 0 (0 %)
Return to work/study	In: 5 (42 %) Out: 7 (58 %) Abstain: 0 (0 %)

### 3.3 The final core outcome set

The final COS included 20 outcomes derived from 208 verbatim responses and 60 outcomes (Table 5). The 12 outcomes that met the criteria for inclusion in the COS from the consensus meeting were added to the eight outcomes, reaching a consensus from the Delphi survey. Both ‘Mortality’ and ‘Survival’ were directly voted ‘Consensus in’ during Delphi round 2. Following discussions regarding the similarity of outputs from this outcome domain, only mortality was retained.

**Table 5:** Final core outcome set for cranioplasty following TBI or stroke. † = outcomes reaching consensus directly from the Delphi.

CORE AREA		OUTCOME DOMAIN	INDIVIDUAL OUTCOME	CONSENSUS VOTES
**Life Impact**	1	Cosmesis	Overall cosmetic outcome following cranioplasty	In: 77 % Out: 23 %
	2	Cosmesis	Patient satisfaction with the cosmetic outcome	In: 85 % Out: 15 %
	3	Function	Overall functional outcome following cranioplasty †	In: 89 % Out: 0
	4	Function	Level of functional independence following cranioplasty †	In: 91 % Out: 0 %
	11	Cognition	The effect of cranioplasty on overall cognition	In: 85 % Out: 15 %
	16	Cognition	The effect of cranioplasty on communication and language †	In: 77 % Out: 0 %
	17	Psychological	The effect that a cranioplasty may have on the overall mental health/well-being of a patient	In: 77 % Out: 23 %
	20	Quality of life	The impact a cranioplasty has on a patient's quality of life †	In: 91 % Out: 0 %
**Pathophysiological**	21	Complications	Overall complications †	In: 83 % Out: 2 %
	22	Complications	Infection †	In: 85 % Out: 0 %
	23	Complications	Wound/soft tissue related issue	In: 83 % Out: 17 %
	24	Complications	Intra-cranial haematoma	In: 100 % Out: 0 %
	26	Complications	Seizure †	In: 75 % Out: 0 %
	27	Complications	Hydrocephalus	In: 91 % Out: 9 %
	28	Complications	Graft specific complications	In: 100 % Out: 0 %
	34	Radiological	Bone resorption	In: 85 % Out: 15 %
	40	Neurological	Any change in the level of consciousness	In: 100 % Out: 0 %
**Resource use**	47	Cranioplasty procedure related	Timing of procedure	In: 83 % Out: 17 %
	48	Cranioplasty procedure related	Any repeat interventions	In: 75 % Out: 25 %
**Mortality**	55	Mortality	Mortality †	In: 85 % Out: 0 %

## 4 Discussion

COAST COS provides a set of outcomes for cranioplasty following TBI or stroke. A mixed methodology approach informed by consensus guidelines (Williamson et al., 2017), defined in a published protocol (Mee et al., 2023b) with transparent reporting throughout, and guided by an international steering committee, resulted in a COS which encompassed the views of patients, relatives, healthcare providers, and researchers. Involvement from all stakeholder groups has ensured a relevant and wide-ranging set of outcomes that make up the final COS. Using an adapted version of the OMERACT 2.0 framework (Tugwell et al., 2007) allowed for clear delineation of outcomes that cover four key areas of clinical practice and research: life impact, pathophysiological manifestations, resource or economic use, and mortality. As a result, the COAST COS is a valid set of outcome domains that can guide clinicians and researchers to the minimum that should be measured in cranioplasty-related research and clinical service provision. Although the COS reflects the outcomes most important to all stakeholders, it acts as a guide for outcome measurement and is intended to answer the question 'what to measure', only and not 'how to measure'. Thus, this COS could be considered a reference for future research, clinical monitoring, and guideline design and implementation, with the hope that its adoption leads to improved consistency in outcome collection and reporting.

One of the difficulties in measuring outcomes related to cranioplasty is differentiating between outcomes specific to cranioplasty and those related to overall recovery from the underlying brain injury. The two are inherently linked, but this COS relates specifically to the outcomes of cranioplasty rather than the overall recovery. Cranioplasty often influences the trajectory of recovery, and there are likely outcomes that could be influenced by cranioplasty but relate more to the overall recovery. It may sometimes be necessary to combine this with a more detailed analysis of the outcome data, ensuring that outcomes relating specifically to cranioplasty are analysed separately as well as combined, where appropriate, with outcomes relating to the overall trajectory of recovery. This is why it is essential to differentiate between 'what to measure’ and 'how to measure’ when considering the implementation of this COS. An example of utilisation could be within registries, for example may help with standardising outcome reporting.

Outcomes following cranioplasty encompass neurological recovery, functional independence, cognitive function, surgical complications, and patient-reported quality of life, with significant variability in assessment methodologies. Functional status may be evaluated using the modified Rankin Scale (mRS), Glasgow Outcome Scale (GOS), or Barthel Index, each possessing distinct measurement characteristics. Cognitive outcomes are assessed inconsistently, ranging from bedside screening to neuropsychological testing, often lacking clearly defined endpoints. Quality of life assessments employ instruments such as EQ-5D, SF-36, or non-validated local tools, resulting in inconsistency across studies. The reporting of complications varies widely, with key adverse events such as infection, hydrocephalus, seizure recurrence, and bone flap resorption being inconsistently defined or omitted. For instance, "wound dehiscence" may refer to superficial skin separation in one report and deep surgical site infection in another. Complication rates range from below 10 % to over 40 %, reflecting methodological rather than biological variability. This heterogeneity limits the comparability of studies, reduces the validity of pooled analyses, and complicates center benchmarking, while hindering conclusions regarding outcome predictors, intervention effectiveness, and complication burden.

A well-defined COS for cranioplasty would help specify what outcomes to collect and would support integration with electronic health records and national registries, such as the German (Sauvigny et al., 2022) and 10.13039/100007472UK (Fountain et al., 2021) registries, both of which have reported outcomes in recent publications. The COS would also help streamline prospective data capture and reducing administrative burden. By aligning research with clinical audit and quality improvement frameworks, a COS also serves to enhance clinical governance and patient-centred care.

### 4.1 Life impact

With 8/20 (40 %) outcome domains that relate to 'life impact’, stakeholders placed great importance on how cranioplasty directly affects the patient. Overall function and functional independence were voted on from the Delphi rounds, and functional outcomes were further discussed in the consensus meeting with the importance of differentiating between global functional outcomes and functional independence. At a glance, these appear the same, but have significant differences. The Extended Glasgow Outcome Scale is a well-established primary outcome measure of the overall functional outcome recommended for TBI (Wilde et al., 2010) and a secondary outcome for stroke (Saver et al., 2012). However, the physical or cognitive gains that may result from cranioplasty can sometimes be more granular and not necessarily result in a substantial change in the overall functional outcome but can still have an important impact on independence, which may have an equally significant impact on the quality of life. Thus, it was clear from voting that these two functional measures should be viewed separately when collecting outcomes.

#### 4.1.1 Cognition

Cognition is a multifaceted variable, with significant challenges in terms of accurate and consistent measurements. The impact of cranioplasty on overall cognition has been voted into the COS, which reflects the growing body of evidence highlighting the impact of cranioplasty on improving cognitive recovery (Stefano et al., 2015; Agner et al., 2002). During the discussions, it was recognised that this outcome domain does not easily translate into a measurable metric, and further work is required to standardise what should be meant by cognition in this context. The authors suggest that overall cognition can be measured using a validated cognitive outcome measurement tool.

#### 4.1.2 Cosmesis

Cosmetic outcomes are subjective and pose a significant challenge to the outcome collection. The clinical implementation of existing clinical scores, such as that developed by cHenker et al. (2018), is important but can be challenging to implement in practice. Nevertheless, 82 % of the patient/relative stakeholder group felt that patient satisfaction with cosmetic outcomes was critically important, and 64 % of them felt that the overall post-cranioplasty cosmesis was critically important (Appendix 3). Both outcome domains were included in the COS, which reflects the importance of cosmesis from both the patient and surgical perspectives. The outcome collection should reflect this emphasis, and future cosmetic outcome measurement tools should include both domains.

### 4.2 Pathophysiological manifestations

#### 4.2.1 Complications

Cranioplasty is associated with a significant burden of complications, reaching up to 45 % (Malcolm et al., 2016), with significant implications for patient outcomes and quality of life. As expected, the outcome domains under the OMERACT core area ‘pathophysiological manifestation” comprised the predominant cranioplasty complications. Future studies should consider including all the complication outcome domains in the COS as a minimum, but development of a tool to aid in data collection to capture these was not part of the scope of this project and should be considered individually for each service or research project.

### 4.3 Resource/economic

#### 4.3.1 Procedure timing

Although time is not strictly an outcome, it was debated at length during the consensus meetings. Cranioplasty timing relates to the effect of the time elapsed from the removal of the cranial bone flap to cranioplasty on the outcome. As many outcomes are directly related to the timing of the cranioplasty procedure, this metric was voted into COS, reflecting its importance.

### 4.4 Limitations

This study was conducted in English, thus limiting the international participation of non-English speakers. Many respondents were from the United Kingdom or the United States, but this should not hinder the validity of the COS. Further work is needed to translate outcomes into outcome measures for different population groups, which will be carefully considered in the future. Finally, the survey did not focus on specific outcome measures; therefore, it is necessary to assess which tests are preferred to evaluate standardised outcomes.

### 4.5 Future work

The COAST COS needs to be disseminated widely to allow for full utilisation, which is especially important when considering how the COS may be used to standardise outcome collection and reporting, and how this could feed into national and international registries related to cranioplasty.

Furthermore, the question of ‘how to collect' must also be answered. An outcome measure for cranioplasty would be beneficial in helping researchers build a standardised evidence base to inform cranioplasty best practices and for clinicians to differentiate between outcomes related to the trajectory of overall recovery and those impacted by cranioplasty. In the development of a universal outcome measurement, it is necessary to consider how the outcome measure should differ in different international healthcare settings. However, at the core, the outcomes from this COS should be translatable and will hopefully facilitate such measures.

## 5 Conclusions

The COAST COS has identified 20 outcomes that covers key cranioplasty outcomes, as assessed by international stakeholders, including surgical, medical, rehabilitation, and nursing professionals, as well as patients and their relatives. Future implementation will aid in the standardisation of outcomes and facilitate the development of cranioplasty-specific outcome measures, aiding between-study comparisons and improving the relevance of trial findings to healthcare professionals and patients.

## Data availability

The data produced in this manuscript are available upon reasonable request from the corresponding author.

## Statement of authorship

All authors listed have met the ICMJE criteria for authorship.

## Funding

This project has been funded by the Division of Academic Neurosurgery, 10.13039/501100000735University of Cambridge.

## Declaration of competing interest

The authors declare that they have no known competing financial interests or personal relationships that could have appeared to influence the work reported in this paper.
