# Supplementary material for: A core outcome set for cranioplasty following stroke or traumatic brain injury - The COAST study
Source: Brain Spine. 2025 Jun 1;5:104288. doi: 10.1016/j.bas.2025.104288 (PMC12205645; doi:10.1016/j.bas.2025.104288)
Supplement: Multimedia component 1 [file mmc1.docx]

# Appendix

## Appendix 1

| **Name** | **HelpText** | **Domain Name** |
| --- | --- | --- |
| Overall cosmetic outcome following cranioplasty | This outcome refers to the overall impact a cranioplasty has on patients’ image of themselves including skull alignment and satisfaction | Cosmesis |
| Patient satisfaction of cosmetic outcome | This outcome refers to how satisfied the patients arewith how they look following the cranioplasty procedure | Cosmesis |
| Overall functional independence following cranioplasty | This outcome refers to how a cranioplasty impacts the overall function of the patient. For example, ability to do things (not just walking) | Function |
| Level of functional independence following cranioplasty | This outcome refers to whether the patients’ independence relating to doing things altered following a cranioplasty | Function |
| Motor function following cranioplasty | This outcome refers to whether the patients’ muscles and movement alter following cranioplasty | Function |
| Effect of pain on function | This outcome refers to whether any pain following cranioplasty has an impact on the patients’ ability to function | Function |
| Changes in overall physical health following cranioplasty | This outcome refers to whether the patients’ physical health changes as a result of the cranioplasty procedure | General health |
| Potential change in bladder control following cranioplasty | This outcome refers to whether a cranioplasty has an effect on a patient’s bladder control, including continence | General health |
| Potential change in bowel control following cranioplasty | This outcome refers to whether a cranioplasty has an effect on a patient’s bowel control, including continence | General health |
| Impact of cranioplasty on overall patient well-being | This outcome refers to whether the patients’ general well-being changes following cranioplasty | General health |
| The effect of cranioplasty on global cognition | This outcome refers to whether a cranioplasty has an effect on a patient’s overall cognition including memory, planning and multi-tasking | Cognition |
| The effect of cranioplasty on executive functioning | This outcome refers to whether a cranioplasty changes the patients’ ability to multi-task and plan specifically | Cognition |
| The effect of cranioplasty on memory | This outcome refers to whether a cranioplasty has an effect on a patient’s memory | Cognition |
| The effect of cranioplasty on attention | This outcome refers to whether a cranioplasty has an effect on a patient’s ability to concentrate | Cognition |
| The effect of cranioplasty on orientation | This outcome refers to whether a cranioplasty has an effect on a patient’s orientation in time, place, and/or person? | Cognition |
| The effect of cranioplasty on communication and language | This outcome refers to whether a cranioplasty has an effect on how a patient can communicate, including speech and language | Cognition |
| The effect that a cranioplasty may have on the overall mental health/well-being of a patient | This outcome refers to whether a cranioplasty impacts the mental health of a patient | Psychological |
| The effect of cranioplasty on depression and anxiety | This outcome refers to whether a patient’s mood and/or anxiety levels change following a procedure of cranioplasty | Psychological |
| The impact a cranioplasty has on a patient’s social outcome | This outcome refers to whether a cranioplasty impacts a patient’s social re-integration post trauma/surgery | Psychological |
| The impact a cranioplasty has on a patient's quality of life | This outcome refers to whether a cranioplasty can impact a patient’s overall quality of life | Quality of life |
| Overall complications | This outcome refers to recording overall complications of cranioplasty | Complications |
| Infection | This complication relates to all infections attributable to cranioplasty | Complications |
| Wound/soft tissue related issue | This complication relates to difficulties or problems concerning to the wound and skin around the cranioplasty site | Complications |
| Intra-cranial haematoma | Includes all types of bleeding within the skull following cranioplasty | Complications |
| Extra cranial haematoma collections | Includes to all types of bleeding outside the skull following cranioplasty | Complications |
| Seizure | This relates to all types of seizures following cranioplasty | Complications |
| Hydrocephalus | This relates to fluid build-up in and around the brain following cranioplasty | Complications |
| Graft specific complications | This includes all issues and problems regarding the cranioplasty graft(either bone flap or synthetic material) | Complications |
| Complications related to the temporal muscle | This concerns issues or problems relating to the muscle/s that lie over the cranioplasty | Complications |
| Medical - systemic complications post operative | This includes all medical complications following a cranioplasty | Complications |
| Cerebrovascular event (stroke) | This relates to any type of stroke that a patient may have as a result of a cranioplasty | Complications |
| Pain - scalp | This relates to any change in pain that the patient has as a result of cranioplasty | Complications |
| Headache | This relates to headaches that the patient may develop following cranioplasty | Complications |
| Bone resorption | This relates to whether the bone plate/flap undergoes reabsorption under the scalp when reviewed on a radiological imaging following cranioplasty | Radiological |
| Bone necrosis | This relates to breakdown of the bone flap that may be seen on a radiological scan following cranioplasty | Radiological |
| Pneumocephalus | This relates to the presence of air within the cranium (skull) seen on radiological imaging following cranioplasty | Radiological |
| Intra cranial haematoma | This relates to all types of bleeding within the skull seen on radiological imaging following cranioplasty | Radiological |
| Cerebral blood flow | This relates to changes of cerebral blood flow (blood vessels in the brain) following cranioplasty | Radiological |
| Osseointegration | This relates to the presence of any bone over growth into the synthetic cranial plate seen on radiological imaging following cranioplasty | Radiological |
| Level of consciousness | This relates to how alert/awake the patient is following cranioplasty compared to before the procedure | Neurological |
| Muscle function | This relates to how the patients’ muscles work and move following cranioplasty compared to before the procedure | Neurological |
| Patients’ ability to swallow and communicate | This relates to whether the cranioplasty has an effect on a patient’s ability to swallow or communicate | Neurological |
| Scalp sensory disturbance | This relates to any change in sensation around the scalp and/or the surgical wound following cranioplasty | Neurological |
| Sleep | This relates to whether the cranioplasty impacts a patient’s ability to sleep or their sleep pattern | Neurological |
| Physical symptoms of neurological nature | This relates to possible changes in a patient’s physical state for example dizziness, buzzing, irritability following cranioplasty | Neurological |
| Patient's co-morbidities | This relates to any co-morbidities (other illnesses) a patient may have and how these may impact a patient’s recovery following cranioplasty | Co-morbidities |
| Timing of procedure | This relates to the timing of the cranioplasty procedure | Cranioplasty procedure related |
| Any repeat interventions | This relates to any interventions following the cranioplasty that may occur, including revision or implant/bone flap removal | Cranioplasty procedure related |
| Implant failure | This relates to recording when the cranioplasty does not work properly and has to be revised or removed | Cranioplasty procedure related |
| Length of intensive care stay | This relates to how long the patient is in intensive care following the cranioplasty operation | Hospital stay |
| Length of hospitalisation | This relates to how long the patient is in hospital for the cranioplasty operation | Hospital stay |
| Return to work/study | This relates to whether the cranioplasty impacts on a patient’s ability to be able to return to work or study | Vocational rehabilitation |
| Cranioplasty costs | This relates to recording the cost of performing a cranioplasty, either as an overall cost or as a breakdown including operating, cost of plate etc | Costs |
| Health economic evaluation | This relates to the overall cost of cranioplasty and its impact on the efficiency and effectiveness of health delivery from an economic perspective | Costs |
| Mortality | This refers to the number of patients that pass away from a cranioplasty-related issue | Mortality |
| Survival | This refers to the average survival rate of patients following cranioplasty | Survival |

## Appendix 2

Outcomes voted in by the steering committee for inclusion in Round 2 of Delphi.

| Life impact | Impact cranioplasty has on patient's engagement and participation in rehabilitation. |
| --- | --- |
|  | The impact cranioplasty has on information processing speed |
|  | The impact cranioplasty has on the quality of life of family members and/or carers. |
| Pathophysiological | Impact cranioplasty has on postural instability and/or balance problems. |

## Appendix 3

| **CORE AREA** |  | **OUTCOME DOMAIN** | **INDIVIDUAL OUTCOME** |
| --- | --- | --- | --- |
| **Life Impact** | 3 | Function | Overall functional outcome following cranioplasty |
|  |  |  |  |
|  | 4 | Function | Level of functional independence following cranioplasty |
|  |  |  |  |
|  | 16 | Cognition | The effect of cranioplasty on communication and language |
|  |  |  |  |
|  | 20 | Quality of life | The impact a cranioplasty has on patient's quality of life |
|  |  |  |  |
| **Pathophysiological** | 21 | Complications | Overall complications |
|  |  |  |  |
|  | 22 | Complications | Infection |
|  |  |  |  |
|  | 26 | Complications | Seizure |
|  |  |  |  |
| **Mortality** | 55 | Mortality | Mortality |
|  |  |  |  |
|  | 56 | Survival | Survival |
|  |  |  |  |

Included Outcomes following 2 rounds of the Delphi Survey

## Appendix 4

Excluded Outcomes following 2 rounds of the Delphi Survey

| **CORE AREA** |  | **OUTCOME DOMAIN** | **INDIVIDUAL OUTCOME** |
| --- | --- | --- | --- |
| **Pathophysiological** | 29 | Complications | Complications related to the temporal muscle |
|  |  |  |  |
|  | 36 | Radiological | Pneumocephalus |
|  |  |  |  |
|  | 38 | Radiological | Cerebral blood flow |
|  |  |  |  |
|  | 39 | Radiological | Osteointegration |
|  |  |  |  |
|  | 43 | Neurological | Scalp sensory disturbance |
|  |  |  |  |
| **Resource use** | 50 | Hospital stay | Length of intensive-care stay |
|  |  |  |  |
|  | 53 | Costs | Cranioplasty costs |
|  |  |  |  |
|  | 54 | Costs | The effect of cranioplasty on the cost to the health system as a whole; known as a health economic evaluation. |
|  |  |  |  |

## Appendix 5

Full score breakdown from Delphi Study

### Life Impact

|  |  |  | **Nurse** | | | **Rehab** | | | **Surgeon** | | | **Patients and/or relatives** | | |
| --- | --- | --- | --- | --- | --- | --- | --- | --- | --- | --- | --- | --- | --- | --- |
|  |  |  | 1,2,3 (%) | 4,5,6  (%) | 7,8,9 (%) | 1,2,3 (%) | 4,5,6 (%) | 7,8,9  (%) | 1,2,3 (%) | 4,5,6  (%) | 7,8,9 (%) | 1,2,3  (%) | 4,5,6  (%) | 7,8,9  (%) |
| Cosmesis | Overall cosmetic outcome following cranioplasty | Rd 1 | 10 | 63 | 23 | 3 | 57 | 41 | 3 | 39 | 59 | 0 | 19 | 63 |
|  |  | Rd 2 | 0 | 74 | 32 | 0 | 41 | 59 | 0 | 36 | 64 | 0 | 27 | 64 |
| Cosmesis | Patient satisfaction of cosmetic outcome | Rd 1 | 0 | 63 | 33 | 0 | 57 | 43 | 1 | 31 | 67 | 0 | 13 | 75 |
|  |  | Rd 2 | 0 | 58 | 42 | 0 | 48 | 52 | 0 | 30 | 70 | 0 | 18 | 82 |
| Function | Overall functional outcome following cranioplasty | Rd 1 | 3 | 20 | 73 | 0 | 14 | 86 | 4 | 14 | 81 | 0 | 13 | 81 |
|  |  | Rd 2 | 0 | 16 | 84 | 0 | 7 | 93 | 0 | 12 | 88 | 0 | 9 | 91 |
| Function | Level of functional independence following cranioplasty | Rd 1 | 3 | 17 | 77 | 0 | 5 | 95 | 4 | 21 | 74 | 0 | 13 | 81 |
|  |  | Rd 2 | 0 | 11 | 89 | 0 | 3 | 97 | 0 | 14 | 86 | 0 | 9 | 91 |
| Function | Motor function following cranioplasty | Rd 1 | 3 | 33 | 53 | 0 | 14 | 86 | 4 | 31 | 64 | 0 | 19 | 75 |
|  |  | Rd 2 | 0 | 37 | 63 | 0 | 10 | 90 | 0 | 18 | 82 | 0 | 9 | 91 |
| Function | Effect of pain on function | Rd 1 | 3 | 47 | 43 | 0 | 27 | 73 | 9 | 54 | 37 | 6 | 13 | 69 |
|  |  | Rd 2 | 0 | 47 | 53 | 0 | 31 | 69 | 2 | 56 | 42 | 0 | 18 | 73 |
| General health | Changes in overall physical health following cranioplasty | Rd 1 | 3 | 47 | 47 | 0 | 43 | 57 | 10 | 31 | 59 | 0 | 25 | 69 |
|  |  | Rd 2 | 0 | 47 | 53 | 0 | 34 | 66 | 2 | 32 | 66 | 9 | 36 | 55 |
| General health | Potential change in bladder control following cranioplasty | Rd 1 | 7 | 53 | 30 | 3 | 38 | 57 | 14 | 56 | 26 | 0 | 31 | 50 |
|  |  | Rd 2 | 0 | 63 | 37 | 0 | 34 | 55 | 16 | 56 | 26 | 9 | 36 | 45 |
| General health | Potential change in bowel control following cranioplasty | Rd 1 | 3 | 57 | 30 | 5 | 38 | 54 | 16 | 56 | 24 | 0 | 31 | 50 |
|  |  | Rd 2 | 0 | 58 | 42 | 11 | 45 | 55 | 18 | 52 | 28 | 9 | 36 | 45 |
| General health | Impact of cranioplasty on overall patient well-being | Rd 1 | 0 | 33 | 63 | 0 | 16 | 81 | 3 | 24 | 73 | 13 | 13 | 69 |
|  |  | Rd 2 | 0 | 42 | 58 | 0 | 38 | 79 | 0 | 16 | 84 | 9 | 18 | 73 |
| Cognition | The effect of cranioplasty on overall cognition. | Rd 1 | 7 | 20 | 67 | 0 | 11 | 89 | 4 | 29 | 67 | 0 | 31 | 63 |
|  |  | Rd 2 | 0 | 21 | 79 | 0 | 10 | 90 | 0 | 16 | 84 | 0 | 36 | 55 |
| Cognition | The effect of cranioplasty on executive functioning | Rd 1 | 3 | 30 | 60 | 0 | 19 | 81 | 4 | 29 | 64 | 0 | 31 | 63 |
|  |  | Rd 2 | 0 | 32 | 63 | 0 | 21 | 79 | 0 | 18 | 78 | 0 | 36 | 55 |
| Cognition | The effect of cranioplasty on memory | Rd 1 | 3 | 37 | 53 | 0 | 24 | 76 | 4 | 39 | 53 | 0 | 31 | 63 |
|  |  | Rd 2 | 0 | 37 | 58 | 0 | 21 | 79 | 0 | 26 | 70 | 0 | 36 | 55 |
| Cognition | The effect of cranioplasty on attention | Rd 1 | 3 | 33 | 57 | 0 | 22 | 78 | 4 | 37 | 54 | 0 | 31 | 63 |
|  |  | Rd 2 | 0 | 42 | 53 | 0 | 17 | 83 | 0 | 26 | 70 | 0 | 36 | 55 |
| Cognition | The effect of cranioplasty on orientation | Rd 1 | 7 | 33 | 53 | 0 | 24 | 76 | 4 | 36 | 56 | 0 | 38 | 56 |
|  |  | Rd 2 | 0 | 37 | 58 | 0 | 24 | 76 | 2 | 24 | 70 | 0 | 45 | 45 |
| Cognition | The effect of cranioplasty on communication and language | Rd 1 | 3 | 23 | 63 | 0 | 22 | 78 | 3 | 33 | 60 | 0 | 38 | 56 |
|  |  | Rd 2 | 0 | 21 | 74 | 0 | 14 | 86 | 0 | 20 | 76 | 0 | 27 | 73 |
| Psychological | The effect that a cranioplasty may have on the overall mental health/well-being of a patient | Rd 1 | 0 | 30 | 67 | 3 | 19 | 78 | 1 | 26 | 73 | 0 | 19 | 75 |
|  |  | Rd 2 | 5 | 26 | 68 | 0 | 24 | 76 | 0 | 14 | 86 | 0 | 18 | 82 |
| Psychological | The effect of cranioplasty on depression and anxiety | Rd 1 | 0 | 47 | 47 | 3 | 24 | 73 | 3 | 39 | 59 | 0 | 31 | 56 |
|  |  | Rd 2 | 5 | 37 | 58 | 5 | 21 | 76 | 0 | 38 | 62 | 0 | 27 | 64 |
| Psychological | The impact a cranioplasty has on a patient’s social outcome | Rd 1 | 0 | 33 | 60 | 0 | 30 | 70 | 3 | 29 | 67 | 6 | 31 | 56 |
|  |  | Rd 2 | 5 | 32 | 63 | 0 | 17 | 83 | 0 | 24 | 74 | 9 | 27 | 55 |
| Quality of life | The impact a cranioplasty has on a patient's quality of life | Rd 1 | 0 | 17 | 80 | 0 | 14 | 86 | 0 | 19 | 81 | 0 | 13 | 81 |
|  |  | Rd 2 | 0 | 11 | 89 | 0 | 7 | 93 | 0 | 0 | 100 | 0 | 18 | 82 |

### Pathophysiological manifestations

|  |  |  | **Nurse** | 30 round 1 participants, 19 round 2 participants | | **Rehab** | 37 round 1 participants, 29 round 2 participants | | **Surgeon** | 70 round 1 participants, 50 round 2 participants | | **Patients and/or relatives** | 16 round 1 participants, 11 round 2 participants | | **Total round 1 and 2** | 153 round 1 participants, 109 round 2 participants | |  |
| --- | --- | --- | --- | --- | --- | --- | --- | --- | --- | --- | --- | --- | --- | --- | --- | --- | --- | --- |
|  |  | Delphi round | Not Important (1,2,3) (%) | Important (4,5,6) (%) | Critical (7,8,9) (%) | Not Important (1,2,3) (%) | Important (4,5,6) (%) | Critical (7,8,9) (%) | Not Important (1,2,3) (%) | Important (4,5,6) (%) | Critical (7,8,9) (%) | Not Important (1,2,3) (%) | Important (4,5,6) (%) | Critical (7,8,9) (%) | Total Not Important (1,2,3) (%) | Total Important (4,5,6) (%) | Total Critical (7,8,9) (%) |  |
| Complications | Overall complications | Round 1 | 0 | 23 | 67 | 0 | 14 | 86 | 4 | 19 | 77 | 19 | 13 | 56 | 4 | 18 | 75 |  |
|  |  | Round 2 | 0 | 21 | 79 | 0 | 10 | 90 | 0 | 10 | 90 | 9 | 9 | 73 | 1 | 12 | 86 |  |
| Complications | Infection | Round 1 | 0 | 20 | 67 | 0 | 19 | 76 | 4 | 3 | 93 | 19 | 13 | 63 | 4 | 11 | 80 |  |
|  |  | Round 2 | 0 | 16 | 84 | 0 | 17 | 83 | 0 | 0 | 100 | 9 | 18 | 73 | 1 | 9 | 90 |  |
| Complications | Wound/soft tissue related issue | Round 1 | 7 | 40 | 40 | 0 | 38 | 54 | 3 | 13 | 84 | 25 | 13 | 56 | 5 | 24 | 65 |  |
|  |  | Round 2 | 0 | 42 | 58 | 0 | 38 | 59 | 0 | 10 | 90 | 18 | 27 | 55 | 2 | 25 | 72 |  |
| Complications | Intracranial haematoma | Round 1 | 0 | 27 | 50 | 0 | 22 | 73 | 6 | 16 | 79 | 19 | 6 | 56 | 5 | 18 | 69 |  |
|  |  | Round 2 | 0 | 21 | 79 | 0 | 21 | 79 | 0 | 4 | 96 | 18 | 9 | 55 | 2 | 12 | 84 |  |
| Complications | Extracranial haematoma  collections | Round 1 | 3 | 23 | 47 | 0 | 41 | 54 | 4 | 37 | 59 | 19 | 6 | 44 | 5 | 32 | 54 |  |
|  |  | Round 2 | 0 | 32 | 63 | 0 | 55 | 45 | 2 | 42 | 56 | 18 | 9 | 55 | 3 | 40 | 54 |  |
| Complications | Seizure | Round 1 | 3 | 23 | 57 | 0 | 30 | 65 | 4 | 34 | 61 | 13 | 6 | 69 | 4 | 28 | 62 |  |
|  |  | Round 2 | 0 | 26 | 74 | 0 | 17 | 83 | 2 | 26 | 72 | 9 | 0 | 73 | 2 | 21 | 75 |  |
| Complications | Hydrocephalus | Round 1 | 0 | 27 | 57 | 0 | 19 | 78 | 3 | 13 | 84 | 6 | 6 | 56 | 2 | 16 | 75 |  |
|  |  | Round 2 | 0 | 21 | 79 | 0 | 10 | 90 | 0 | 10 | 90 | 18 | 0 | 64 | 2 | 11 | 85 |  |
| Complications | Graft specific  complications | Round 1 | 0 | 30 | 43 | 0 | 38 | 54 | 4 | 27 | 67 | 19 | 7 | 50 | 4 | 28 | 58 |  |
|  |  | Round 2 | 0 | 26 | 74 | 0 | 28 | 66 | 0 | 18 | 82 | 18 | 9 | 64 | 2 | 21 | 74 |  |
| Complications | Complications related  to the temporal muscle | Round 1 | 3 | 33 | 40 | 0 | 59 | 35 | 13 | 60 | 27 | 13 | 19 | 38 | 8 | 50 | 33 |  |
|  |  | Round 2 | 0 | 47 | 42 | 0 | 59 | 38 | 6 | 70 | 24 | 18 | 18 | 45 | 5 | 58 | 33 |  |
| Complications | Medical –  systemic complications  post operative | Round 1 | 0 | 33 | 40 | 3 | 43 | 51 | 11 | 47 | 40 | 19 | 13 | 50 | 8 | 40 | 44 |  |
|  |  | Round 2 | 0 | 53 | 47 | 5 | 45 | 52 | 4 | 54 | 40 | 18 | 18 | 64 | 5 | 48 | 47 |  |
| Complications | Cerebrovascular event  (stroke) | Round 1 | 0 | 7 | 77 | 3 | 19 | 73 | 10 | 23 | 67 | 13 | 13 | 56 | 7 | 18 | 69 |  |
|  |  | Round 2 | 0 | 5 | 95 | 0 | 17 | 83 | 2 | 12 | 86 | 9 | 18 | 64 | 2 | 13 | 84 |  |
| Complications | Pain – scalp | Round 1 | 7 | 57 | 27 | 0 | 51 | 43 | 13 | 54 | 33 | 25 | 19 | 50 | 10 | 50 | 36 |  |
|  |  | Round 2 | 0 | 68 | 32 | 0 | 59 | 41 | 6 | 64 | 30 | 9 | 36 | 55 | 4 | 61 | 36 |  |
| Complications | Headache | Round 1 | 0 | 67 | 23 | 0 | 49 | 46 | 13 | 50 | 37 | 19 | 25 | 50 | 8 | 50 | 38 |  |
|  |  | Round 2 | 0 | 68 | 32 | 0 | 45 | 55 | 4 | 60 | 36 | 9 | 18 | 73 | 3 | 53 | 44 |  |
| Radiological | Bone resorption | Round 1 | 13 | 13 | 20 | 14 | 43 | 30 | 13 | 31 | 54 | 0 | 6 | 31 | 12 | 28 | 39 |  |
|  |  | Round 2 | 5 | 42 | 26 | 26 | 41 | 21 | 8 | 32 | 60 | 9 | 9 | 27 | 13 | 34 | 40 |  |
| Radiological | Bone necrosis | Round 1 | 7 | 13 | 27 | 14 | 24 | 46 | 17 | 19 | 46 | 17 | 19 | 59 | 14 | 19 | 43 |  |
|  |  | Round 2 | 5 | 21 | 47 | 21 | 28 | 41 | 6 | 22 | 70 | 9 | 0 | 36 | 10 | 21 | 55 |  |
| Radiological | Pneumocephalus | Round 1 | 17 | 17 | 13 | 16 | 46 | 24 | 33 | 44 | 23 | 0 | 6 | 19 | 22 | 35 | 21 |  |
|  |  | Round 2 | 16 | 42 | 16 | 26 | 55 | 10 | 36 | 44 | 20 | 9 | 18 | 18 | 28 | 44 | 17 |  |
| Radiological | Intra cranial haematoma | Round 1 | 3 | 20 | 37 | 3 | 19 | 68 | 9 | 23 | 69 | 0 | 6 | 44 | 5 | 20 | 59 |  |
|  |  | Round 2 | 0 | 11 | 74 | 5 | 14 | 79 | 4 | 12 | 84 | 0 | 9 | 55 | 3 | 12 | 78 |  |
| Radiological | Cerebral blood flow | Round 1 | 3 | 17 | 37 | 5 | 35 | 43 | 16 | 34 | 49 | 0 | 6 | 38 | 9 | 28 | 44 |  |
|  |  | Round 2 | 0 | 26 | 53 | 5 | 14 | 79 | 6 | 44 | 50 | 0 | 9 | 36 | 4 | 29 | 57 |  |
| Radiological | Osteointegration | Round 1 | 3 | 23 | 17 | 14 | 43 | 19 | 21 | 43 | 36 | 0 | 13 | 31 | 14 | 36 | 27 |  |
|  |  | Round 2 | 0 | 42 | 16 | 16 | 59 | 10 | 18 | 50 | 32 | 0 | 18 | 36 | 13 | 48 | 24 |  |
| Neurological | Any change in level of  consciousness  (how awake the patient is)  following cranioplasty  compared to before | Round 1 | 7 | 17 | 70 | 3 | 11 | 84 | 4 | 19 | 84 | 13 | 31 | 50 | 5 | 18 | 78 |  |
|  |  | Round 2 | 0 | 26 | 74 | 0 | 10 | 90 | 0 | 14 | 86 | 0 | 36 | 64 | 0 | 17 | 83 |  |
| Neurological | Muscle function | Round 1 | 3 | 37 | 50 | 0 | 43 | 57 | 19 | 34 | 46 | 0 | 25 | 69 | 9 | 36 | 52 |  |
|  |  | Round 2 | 0 | 47 | 53 | 0 | 41 | 59 | 8 | 36 | 56 | 0 | 27 | 73 | 4 | 39 | 58 |  |
| Neurological | Patient’s ability to  swallow and communicate | Round 1 | 3 | 27 | 63 | 0 | 16 | 84 | 9 | 37 | 53 | 6 | 19 | 69 | 5 | 28 | 64 |  |
|  |  | Round 2 | 0 | 42 | 58 | 0 | 10 | 90 | 2 | 36 | 62 | 9 | 18 | 73 | 2 | 28 | 70 |  |
| Neurological | Scalp sensory disturbance | Round 1 | 13 | 60 | 13 | 11 | 57 | 32 | 34 | 53 | 13 | 19 | 31 | 50 | 23 | 53 | 22 |  |
|  |  | Round 2 | 5 | 84 | 11 | 11 | 69 | 24 | 26 | 60 | 14 | 9 | 36 | 45 | 17 | 64 | 19 |  |
| Neurological | Sleep | Round 1 | 7 | 57 | 27 | 3 | 38 | 59 | 7 | 53 | 37 | 0 | 50 | 50 | 5 | 50 | 42 |  |
|  |  | Round 2 | 5 | 53 | 42 | 0 | 34 | 66 | 4 | 46 | 46 | 0 | 27 | 73 | 3 | 42 | 53 |  |
| Neurological | Physical symptoms of  neurological nature | Round 1 | 0 | 47 | 37 | 0 | 41 | 59 | 1 | 50 | 49 | 6 | 31 | 56 | 1 | 45 | 50 |  |
|  |  | Round 2 | 0 | 68 | 32 | 0 | 41 | 59 | 0 | 48 | 52 | 9 | 18 | 64 | 1 | 47 | 51 |  |
| Co-morbidities | Patient's co-morbidities | Round 1 | 0 | 60 | 27 | 0 | 59 | 41 | 4 | 40 | 56 | 6 | 31 | 50 | 3 | 48 | 46 |  |
|  |  | Round 2 | 0 | 74 | 26 | 5 | 66 | 31 | 0 | 50 | 50 | 9 | 18 | 64 | 2 | 55 | 42 |  |

### Resource use

|  |  |  | **Nurse** | 30 round 1 participants, 19 round 2 participants |  | **Rehab** | 37 round 1 participants, 29 round 2 participants |  | **Surgeon** | 70 round 1 participants, 50 round 2 participants |  | **Patients and/or relatives** | 16 round 1 participants, 11 round 2 participants |  | **Total round 1 and 2** | 153 round 1 participants, 109 round 2 participants |  |
| --- | --- | --- | --- | --- | --- | --- | --- | --- | --- | --- | --- | --- | --- | --- | --- | --- | --- |
|  |  | Delphi round | Not Important (1,2,3) (%) | Important (4,5,6) (%) | Critical (7,8,9) (%) | Not Important (1,2,3) (%) | Important (4,5,6) (%) | Critical (7,8,9) (%) | Not Important (1,2,3) (%) | Important (4,5,6) (%) | Critical (7,8,9) (%) | Not Important (1,2,3) (%) | Important (4,5,6) (%) | Critical (7,8,9) (%) | Total Not Important (1,2,3) (%) | Total Important (4,5,6) (%) | Total Critical (7,8,9) (%) |
| Cranioplasty procedure related | Timing of procedure (how long after the removal of the bone piece) | Round 1 | 3 | 43 | 33 | 5 | 30 | 46 | 1 | 36 | 63 | 6 | 31 | 44 | 3 | 35 | 51 |
|  |  | Round 2 | 5 | 53 | 37 | 5 | 34 | 52 | 0 | 36 | 64 | 0 | 36 | 36 | 2 | 39 | 53 |
| Cranioplasty procedure related | Any repeat interventions | Round 1 | 0 | 40 | 27 | 0 | 35 | 54 | 3 | 13 | 84 | 13 | 6 | 50 | 3 | 23 | 62 |
|  |  | Round 2 | 5 | 37 | 42 | 0 | 34 | 59 | 0 | 6 | 94 | 18 | 9 | 55 | 3 | 19 | 72 |
| Cranioplasty procedure related | Implant failure | Round 1 | 0 | 17 | 50 | 3 | 16 | 68 | 4 | 7 | 89 | 6 | 19 | 44 | 3 | 12 | 71 |
|  |  | Round 2 | 0 | 5 | 89 | 5 | 10 | 76 | 0 | 0 | 100 | 18 | 18 | 45 | 3 | 6 | 86 |
| Hospital stay | Length of intensive care stay | Round 1 | 7 | 57 | 27 | 3 | 51 | 43 | 19 | 40 | 37 | 19 | 19 | 44 | 12 | 44 | 37 |
|  |  | Round 2 | 5 | 68 | 26 | 16 | 55 | 34 | 14 | 42 | 42 | 9 | 55 | 9 | 13 | 51 | 34 |
| Hospital stay | Length of hospitalisation | Round 1 | 7 | 50 | 33 | 3 | 57 | 41 | 14 | 34 | 51 | 13 | 31 | 44 | 10 | 42 | 44 |
|  |  | Round 2 | 0 | 68 | 32 | 5 | 66 | 31 | 8 | 38 | 54 | 0 | 73 | 9 | 5 | 54 | 39 |
| Vocational rehabilitation | Return to work/study | Round 1 | 0 | 37 | 57 | 0 | 27 | 70 | 4 | 19 | 61 | 6 | 38 | 44 | 3 | 26 | 61 |
|  |  | Round 2 | 0 | 58 | 42 | 0 | 14 | 86 | 0 | 20 | 80 | 27 | 27 | 45 | 3 | 26 | 72 |
| Costs | Cranioplasty costs | Round 1 | 17 | 60 | 7 | 11 | 62 | 19 | 10 | 47 | 43 | 25 | 31 | 19 | 13 | 52 | 27 |
|  |  | Round 2 | 21 | 63 | 5 | 21 | 69 | 14 | 8 | 54 | 38 | 18 | 55 | 0 | 15 | 60 | 22 |
| Costs | The effect of cranioplasty on the cost to the health system as a whole; known as a health economic evaluation. | Round 1 | 10 | 57 | 17 | 11 | 57 | 27 | 7 | 46 | 47 | 13 | 38 | 19 | 9 | 50 | 33 |
|  |  | Round 2 | 21 | 53 | 21 | 16 | 66 | 21 | 6 | 48 | 46 | 18 | 55 | 9 | 13 | 54 | 31 |

### Mortality

|  |  |  | **Nurse** | 30 round 1 participants, 19 round 2 participants |  | **Rehab** | 37 round 1 participants, 29 round 2 participants |  | **Surgeon** | 70 round 1 participants, 50 round 2 participants |  | **Patients and/or relatives** | 16 round 1 participants, 11 round 2 participants |  | **Total round 1 and 2** | 153 round 1 participants, 109 round 2 participants |  |
| --- | --- | --- | --- | --- | --- | --- | --- | --- | --- | --- | --- | --- | --- | --- | --- | --- | --- |
|  |  | Delphi round | Not Important (1,2,3) (%) | Important (4,5,6) (%) | Critical (7,8,9) (%) | Not Important (1,2,3) (%) | Important (4,5,6) (%) | Critical (7,8,9) (%) | Not Important (1,2,3) (%) | Important (4,5,6) (%) | Critical (7,8,9) (%) | Not Important (1,2,3) (%) | Important (4,5,6) (%) | Critical (7,8,9) (%) | Total Not Important (1,2,3) (%) | Total Important (4,5,6) (%) | Total Critical (7,8,9) (%) |
| Mortality | Mortality | Round 1 | 3 | 7 | 77 | 0 | 19 | 73 | 9 | 10 | 81 | 6 | 6 | 63 | 5 | 11 | 76 |
|  |  | Round 2 | 0 | 11 | 89 | 0 | 10 | 83 | 0 | 4 | 96 | 0 | 9 | 73 | 0 | 7 | 89 |
| Survival | Survival | Round 1 | 0 | 10 | 80 | 0 | 14 | 76 | 6 | 21 | 70 | 0 | 6 | 81 | 3 | 16 | 75 |
|  |  | Round 2 | 5 | 5 | 84 | 0 | 7 | 90 | 2 | 18 | 80 | 0 | 9 | 82 | 2 | 12 | 83 |
